# Peer pressure and home environment as predictors of disruptive and risky sexual behaviours of secondary school adolescents

**DOI:** 10.4314/ahs.v18i2.4

**Published:** 2018-06

**Authors:** Dorothy Ebere Adimora, Immaculata Nwakaego Akaneme, Eucharia Nchedo Aye

**Affiliations:** Department of Educational Foundations, Faculty of Education, University of Nigeria, Nsukka, Nigeria

**Keywords:** Home, peer group, disruptive, behaviour, adolescents

## Abstract

**Background:**

Not much is understood about the predictive power of home environment and peer pressure on disruptive behaviour and risky sexual behaviour of adolescents.

**Objectives:**

The study investigated the predictive power of home environment and peer pressure on disruptive behaviour and risky sexual behaviour of adolescents in secondary school class two in Enugu State, Nigeria. Four research questions and four null hypotheses guided the study.

**Methods:**

The design was a cross sectional survey of correlational study. The study was carried out in the six Education zones in Enugu State, Nigeria. The population was all the 31,680 senior secondary class two (SS11) adolescents in 285 secondary schools in Enugu State, Nigeria in 2015/2016 academic session. The instruments for data collection were an observation schedule, an interview session and a structured questionnaire of four clusters. To determine the R-squares for each regression model, a multivariate multiple regression model was conducted using “MANOVA” and “MVREG”.

**Results:**

This study indicates adolescents who reported their home environments to be stimulated displayed compliant behavior and none or lowered risky sexual behaviour, whilst those with chaotic and unstimulated home environment displayed disruptive behaviours. It was found that adolescents who show a heightened sensitivity to positive peer pressure demonstrated compliant and no or lowered risky sexual behavior whilst, those that are engaged with negative peer pressure strongly exhibit disruptive and risky sexual behaviour.

**Conclusion:**

Unstimulated home environment and negative peer group could consequently interact to predispose these adolescents to disruptive behaviour and risky sexual behaviour.

## Introduction

Adolescence period is often believed to be a difficult and critical period of transition because of various qualitative shifts prevalent at that stage of life. This stage conflicts with breaking away from the old self and interest of the childhood memories which are accompanied by significant changes of various degrees, such as physical, biological, intellectual, social and emotional developments,^1^ described adolescence as the period of 10–19 years, within the life span when most of a person's biological, cognitive, psychological, and social characteristics are changing from what is typically considered child-like to what is considered adult-like^2^. Most times, adolescents get engulfed in their ambiguous status. Being neither children nor adults, they frequently get themselves involved in conflicts with younger children and adults in their home environment. This is because the home environment produces the first and the most insistent and suitable influence on the allround development of individuals at every stage of their development of which adolescence stage is inclusive.

Home Environment is a descriptive profile which yields a systematic assessment of the caring environment where the child is reared. It refers to aspects of peoples' domestic lives that contribute to their living conditions. These factors may be physical such as poverty, psychological conditions due to parenting and social circumstances. Various home environment factors have been shown to be important such as parents' responsiveness, discipline style, and involvement with the child; organization of the environment; availability of appropriate learning materials; opportunities for daily stimulation. Research has revealed that parents who provide a warm, responsive, and supportive home environment; encourage exploration; stimulate curiosity; and provide play and learning materials, accelerate their children's intellectual development, compliant behaviour and make them less vulnerable to risky sexual behaviour^3^.

According to Onyehalu^4^ the home environment could pose many handicaps or be a source of special advantage in the life of adolescents.^4^ Further opined that a poor or an impoverished home environment may adversely influence the child's effectiveness in the society. Cohen^5^ argues that adolescents from poor home environments engage in early sexual relationships, thus are vulnerable to HIV/AIDS because they lack proper care and access to methods for practicing safer sex, engage in informal prostitution because they are less empowered economically, legally, culturally, and socially compared with those from a comfortable home. Generally, adolescents seem to engage in risky sexual behaviour (RSB) due to economic needs, unresponsive parents, relatives and elder siblings that engage in RSB whom they model. Furthermore, adolescents from impoverished home environment in Nigeria, seem to lack intellectual and social stimulation, have uneducated parents who are so busy, unresponsive and inaccessible to their children. Some of them also lack requisite knowledge and skills to impart on their children. It is not surprising, therefore, that children from such homes may find it difficult to keep up with those who live in healthier and more stimulating home environments. It is then possible that some adolescents from impoverished home environments might desire to follow the crowd and do what majority of those from healthier and more stimulated home environments do, which could predispose them to activities which they would not have ordinarily engaged in.

However, research has also shown that adolescents from one-parent families or impoverished home environments are more likely to demonstrate increased substance and alcohol use as well as more emotional problems, such as depression and loneliness, compared to those in an enhanced or stimulated home environment^6^. Furthermore, children or adolescents from such impoverished and disorganized home environments seem more likely to develop risky sexual behaviour. For example, a study by Moore^7^ reported that a home environment with disruption of parents' marriage and a situation where the adolescent is living with a single parent is related with risky sexual behaviour, because there is seemingly less monitoring of the adolescent.

According to Omeje^8^ sexual behaviour is an individual's experience or expression of sexual feelings. It includes passionate kissing, fondling, petting, oral-to-anal stimulation and hand-to-genital stimulation (includes “making out”, “dry sex/humping”, “fingering”, analingus, “rimming”. Risky sexual behaviour (RSB) is any sexual activity that increases the risk of contracting sexually transmitted infection or becoming pregnant. RSB includes having sex at an early age, having multiple sexual partners, having sex while under the influence of alcohol or drugs, and unprotected sexual behaviour^9^.

RSBs are those behaviours that could lead contributors to death, disability, and social problems, such as tobacco use, unhealthy dietary behaviours, inadequate physical activity, alcohol and other drug use, unintended pregnancy and sexually transmitted diseases (including HIV), and behaviours that contribute to unintentional injuries and violence.

The complex influence of home environment begins before birth and is carried into transition to adult independence and pursuit of individual identity. The home environment where children grow up can have an immense impact on whether or not they develop the behavioural patterns that define disruptive behaviour disorder (DBD). Research has shown that family instability, such as parental divorce or frequent disruption to routines, e.g. frequent change of schools or of child care providers result in disruptive behaviours among children and adolescents^10^. Children raised in chaotic homes- characterized by noise, overcrowding, and a lack of order-tend to score lower on tests of cognitive ability and self-regulatory capabilities, have poorer language abilities, and score higher on measures of problem behaviours and learned helplessness than do children raised in less chaotic environments^11^,^12^. If children are surrounded by a chaotic home life, they may begin to act out simply because there is no emotional stability, this could give them the leverage to be deviant. Similarly, children who are raised without appropriate discipline or whose parents tend to be more absent than not, can experience major impacts on the ways in which they behave. Atypical mother-child interaction at the time of birth has also been theorized to have an effect on the onset of disruptive behaviour disorder.

However, dysfunctional family relations as well as parental abuse/neglect, poor sibship relationship, poor parenting and family adversity have been associated with development and reinforcement of conduct problems. Parental psychopathology (especially a family history of conduct disorder/antisocial personality and substance use) are also related to an elevated risk for disruptive symptoms in offspring^13^. This is especially true when the adolescents from the impoverished home environments fail to get their basic necessities and are easily influenced by the peers they socialize with; these peers could exert pressure on them to do what they would not have ordinarily done. A study has revealed that during the transition from childhood to adolescence, relationships with peers become increasingly elaborate, more personal and emotional interactions with peers dominate adolescents' social environment, with American adolescents spending more than half of their awake-time with peers^14^.

Peer pressure is influence of a peer group, observers or individual exerts that encourages others to change their attitudes, values, or behaviour to conform to groups. Peers exert a significant influence on behaviour during adolescence^15^. Peer pressure is commonly associated with events of adolescent risk taking (such as delinquency, drug abuse, sexual behaviours, and reckless driving) because these activities commonly occur in the company of peers^16^. The peer group is an important factor in adolescent development and has some bearing on teenagers' decisions about sex. Adolescents (ages 11 to 18) report that they are most likely to get information about sexual health issues from their peers^17^.

However, recent studies have shown that peer pressure might have positive or negative influences. A child influenced by a positive peer pressure can be inspired to be more focused and zealous. Positive peer pressure can help one reflect actions and amend one's ways to become a better individual. It allows one to share experiences and feelings and learn how to resolve conflicts, it provides adolescents with an appropriate environment for healthy development^18^. Conversely, adolescents' negative peer pressure, could lead to negative acts such as smoking, drinking, negative way of dressing and speaking, using illicit substances, engagement in sexual behaviours, adopting and accepting violence, adopting criminal and anti-social behaviours and in many other areas of the adolescent's life^19^. As adolescents begin to spend more time with peers, the relative importance of peer group influence over family influence may change. Influence of peer pressure has been linked to adolescent risky sexual behaviour.

Risky sexual behaviours are common behaviours that increase one's risk of contracting sexually transmitted infections and having unintended pregnancies. In Nigeria, data shows that adolescents 15 to 19 years old engage in high risky sexual behaviour: 56.4% of sexually active boys and 39.6% of sexually active girls had had unprotected sex with non-marital sexual partners in the last 12 months of a survey. The proportion of adolescents who engage in this high risky sexual behaviour was higher than what was observed in other age groups. Other high risky sexual behaviours - transactional sex, multiple sex partnership, mixing of sexual partners - are on the increase among adolescents in Nigeria^20^.

Disruptive behaviour disorders (DBDs) are a group of behavioural disorders defined by ongoing patterns of hostile and defiant behaviours that children and adolescents direct towards any type of authority figure. While all children/adolescents go through periods of testing limits by acting out in negative behaviours, children/adolescents with DBD participate in these behaviours to such an extreme that it affects their everyday life, as well as the lives of those around them^13^.

The two most common forms of disruptive behaviour are oppositional defiant disorder (ODD) and conduct disorder (CD). CD is characterized by persistent and repetitive behaviours that involve violating the basic rights of other human beings and severely breaking rules set to enforce age-appropriate societal norms. Oppositional defiant disorder (ODD) is similar to conduct disorder but usually presents itself earlier in a child's life. ODD is characterized by patterns of hostile, defiant, and disobedient behaviours directed at parents, teachers, and any other type of authority figure^13^.

Adolescents with DB may exhibit behavioural, cognitive and psychological symptoms. Behavioural symptoms are social isolation, bullying, revenge-seeking behaviours, lying, stealing, willful destruction of property, blaming others, actively defying or refusing to comply with rules, being cruel to animals, playing with fire. Cognitive symptoms are difficulty concentrating, frequent frustration, memory impairment, inability to “think before speaking”, lack of problem-solving skills. The psychosocial symptoms are lack of empathy, lack of remorse, false sense of grandiosity, persistent negativity, low self-esteem, chronic annoyance and irritability^14^.

If children do not receive proper treatment interventions, the effects of DB can be long-lasting and can, in some cases, lead to development of anti-social personality disorder. Some examples of the long-term effects that untreated DB can have on a person include: criminal involvement, incarceration, inability to develop and maintain healthy, meaningful relationships, social isolation, substance abuse and risky sexual behaviours^21^.

Adolescents, have been found to be at high risk for having sex at an early age, having multiple sexual partners, having sex while under the influence of alcohol or drugs, and unprotected sexual behaviour, vulnerable to violence and many negative health consequences related to risky sexual behaviours, including infection with human immunodeficiency virus & HIV), other sexually transmitted diseases e.g, syphilis, *Chlamydia*) and several other conditions such as substance use/abuse, internalized disorders (depressive, anxiety disorders), and learning disorders^23^.

Furthermore, during this stage, some adolescents from impoverished home environments seem to be deprived of their basic necessities and are, therefore, vulnerable to relationship with negative peer group which are seemingly increasingly important to them because they want to belong, they are also vulnerable to disruptive behaviour disorder (DBD) such as social isolation, bullying, revenge-seeking behaviours, lying, stealing, willful destruction of properties, blaming others, actively defying or refusing to comply with rules, being cruel to animals, playing with fire, difficulty concentrating, frequent frustration, inability to “think before speaking”, lack of problem-solving skills, lack of empathy and remorse, false sense of grandiosity, persistent negativity, low self-esteem, chronic annoyance and irritability. Frequent risk factors for DBDs are emotional problems, mood disorders, multiple mental disorders and environmental risk factors, family difficulties and substance abuse^22^.

However, it seems Nigeria and particularly Enugu State secondary schools are not yet aware of turbulences of adolescence stage triggered by hormonal changes and some dramatic and drastic developments which are prevalent at this stage of life. These adolescents, if not properly monitored may be influenced negatively to engage in risky sexual behaviour and disruptive behaviour which could be destructive and harmful to their lives. Some adolescents in Enugu State have poor home environments and are consequently, easily influenced by peers, to engage in disruptive and risky sexual behaviours. It has been observed that not much is known about the extent to which home environment and peer pressure predict adolescents' disruptive and risky sexual behaviours in Nigeria. This therefore, makes this study imperative. The problem of this study, therefore, put in a question form is: What are the predictive powers of home environment and peer pressure on disruptive and risky sexual behaviours of SSII adolescents in Enugu State, Nigeria? The general purpose of this study is to ascertain the predictive power of home environment and peer pressure on disruptive behaviour and risky sexual behaviour of secondary school class three adolescents in Enugu State, Nigeria. Specifically, the study sought to: Determine the relationship of peer group pressure on adolescents' disruptive behaviour disorder; Investigate peer group pressure and the predictor power on adolescents' risky sexual behaviour; Investigate the predictive power of home environment on adolescents' disruptive behaviour disorder; Investigate the predictive power of home environment on adolescents' risky sexual behaviour.

## Method

Following approval by University of Nigeria, Nsukka, Ethical Committee for Non-Clinical Research Involving Human Subjects, a cross-sectional analysis of the baseline assessment of a longitudinal study among early adolescents was adopted for the study. The study was carried out in the six education zones in Enugu state, Nigeria: Agbani, Awgu, Enugu, Nsukka, Obollo-Afor and Udi (N = 31,680) all the senior secondary class two adolescents in 285 secondary schools in Enugu State, Nigeria in 2015/2016 academic session. The target population was students in Senior Secondary class II, (SS11) which constitutes one-sixth of the entire student population in Enugu State, Nigeria. A multi-stage sampling technique was employed to draw a sample of 372 secondary school adolescents. An observation schedule and structured interview session and well-structured questionnaire were also used to elicit information on adolescents' DBD whilst the RSB were ascertained based on adolescents' history of sexual behaviour in the past 12 months. This was done through the help of six research assistants. The instruments were validated by experts. The reliability coefficient of the instruments were ascertained by subjection to field trial. The adolescents were asked to complete the questionnaire on their home environment, peer pressure, disruptive behaviour disorder and risky sexual behaviours. A questionnaire with four clusters was also used to elicit information on theadolescents' home environment, peer pressure, risky sexual behaviour and disruptive behaviour disorder.

## Instruments

### Home environment

The adolescents' home environment was ascertained using a 53-item questionnaire divided into eight sub-scales: responsivity, encouragement of maturity, emotional climate, learning materials and opportunities, enrichment, family companionship and physical environment. Participants indicated the extent to which they agreed to the home environment item statements. Statements were responded to using four-point Likert type format: Items included: very much, pretty much, just a little, not at all. Items included: “My family has a good, regular and predictable daily schedule for my home activities” “My family requires me to carry out certain self-care routines such as making bed, cleaning room, cleaning up after spills, and bathing self ” “I do not receive unnecessary physical punishment from my parents” “My family encourages me to imbibe a reading habit”. “My parents monitor the type of movies I and my siblings watch and ensure that I do not watch inappropriate movies” My room has educative pictures or wall decoration which appeal to me”. Home environment scores were calculated by finding the total scores for the items. Internal consistency was originally established (range from 0.85 to 0.92) with a sample of home environment. The test-retest reliability ranged from 0.87 to 0.90 with 3 weeks between tests. For the purpose of this study, reliability showed a good internal consistency of items Cronbach's alpha (*α* = 0.81).

### Peer Group Pressure

A researcher-developed peer group pressure (PGP) scale of 16 items was used to assess the adolescents' peer group influence. The PGP scale is related to the adolescents' tendency to be influenced by their peers. The items stated included: “I enjoy spending most of my time with my peers,” I hate deviating from my friends' instructions”. “I spend most of my time with my friends in reading and doing my homework.” “Sometimes I skip school because my friend(s) skip(s).” Respondents expressed their degree of agreement on a 4-point Likert-type scale of never = 4, seldom = 3, sometimes = 2, and always = 1. Cronbach's alpha reliability coefficient for the scale in this study was 0.81. The scale generally has high reliability: Test-retest correlations are in the range of 0.972 – 0.86. Coefficient alpha was 0.92. The present sample reported a coefficient alpha of α = 0.78. No norms or cut-off were found for non-clinical samples.

### Risky sexual behaviour

The self-reported adolescent version of the student's risky sexual behaviour questionnaire (SRSBQ) is a brief questionnaire aimed at assessing students' risky sexual behaviour within the last 12 months. This questionnaire has 30 items. The item statements include, “within the last 12 months”: “I have engaged in sexual behaviour with a partner even though I did not have sex”, “I had “hooked up” and engaged in sexual behaviour with someone I did not know well”, “I have gotten so drunk or high and could not control my sexual behaviours”, “I had vaginal intercourse without a latex or condom”. Respondents expressed their degree of agreement on a 4-point Likert-type scale of very much = 4, pretty much = 3, just a little = 2, and not at all = 1. Cronbach's alpha reliability coefficient for the scale in this study was 0.79. There was generally a high reliability for the scale. Test-retest correlations ranged from 0.68 – 0.87. Coefficient alpha was 0.94. The present sample reported a coefficient alpha of α = 0.81. No norms or cut-off were found for non-clinical samples.

### Disruptive behaviour disorder

Disruptive behaviour disorder was ascertained using observation schedule and questionnaire. This scale includes 44 items assessing their disruptive behaviour disorder: “I often interrupt or intrude on others (e.g., butt into conversations or games)”, “I often initiate physical fights with other members of my class or household”, “I often initiate physical fights with other members of my class or household”, “I make careless mistakes in school work or other activities”. Respondents responded to the items on a 4-point Likert-type scale of very much = 4, pretty much = 3, just a little = 2, and not at all = 1. Cronbach's alpha reliability coefficient for the scale in this study was 0.81 which was a generally high reliability for the scale. Test-retest correlations ranged from 0.71 – 0.85. Coefficient alpha was 0.89. No norms or cut-off were found for non-clinical samples.

### Data analysis

To study the predictive power, the researchers conducted several regression models. To estimate the statistical significance of the models, the researchers calculated Wilks, Lamba, Pillai's trace, Lawley-Hotelling trace, and Roy's largest roots. Furthermore, to determine the R-squares for each regression model, a multivariate multiple regression model was conducted using the commands “MANOVA” and “MVREG” (UCLA: Statistical Consulting Group, 2016). All analyses were conducted using STATA version 12.1.

### Ethical aspects

This study was approved by the University of Nigeria, Nsukka research committee (October 18^th^, 2016). It was performed in accordance with the Declaration of research committee of the University. All participants gave informed consent and assent.

## Results

In the multivariable multiple regression analyses, all tests to assess the model showed that it was significant (Wilks, lamba, Pillai's trace, Lawley-Hotelling trace, and Roy's largest roots had a p-value < 0.001). A full model including the two independent/predictor variables explained 17%, 15%, of the variance in the outcome variables; home environment and peer pressure respectively. In the adjusted results, it was found that adolescents' home environment was strongly associated with all facets of disruptive behaviour and risky sexual behaviour. Having a stimulated home environment reduced the probability of having adolescents with disruptive behaviour and risky sexual behaviour, whilst unstimulated home environment increased the probability of having disruptive behaviour and risky sexual behaviour. Adolescents who had positive peer pressure, had higher probability of being more focused, zealous, allows one to share experiences, feelings, learn how to resolve conflicts, and provides adolescents with an appropriate environment for healthy development. Conversely, adolescents' negative peer pressure could be associated with negative or disruptive behaviour and disruptive acts such as smoking, drinking, immodest ways of dressing and speaking, using illicit substances, engagement in risky sexual behaviours, adopting and accepting violence, adopting criminal and anti-social behaviours.

### Regression analysis

To test the predictive value of home environment and peer pressure on disruptive behaviour and risky sexual behaviour of adolescents, a multiple linear regression was employed. A graphical examination of the residuals in a QQ plot indicated no departure from normality revealing the data as suitable for regression analysis. Research shows that the predictor variables; home environment and peer pressure of the adolescents significantly predict their disruptive and risky sexual behaviours. A two-step regression models were created to investigate the strength of the predictive variables home environment (responsivity, encouragement of maturity, emotional climate, learning materials and opportunities, enrichment, family companionship and physical environment) and peer pressure as predictors of risky sexual behaviour and disruptive behaviour disorder. None of the assumptions of multiple regression was violated. The results of the analysis are shown in Table 1.

**Table 1:** Multiple regression examining the predictive strength of home environment on risky sexual behaviour and disruptive behaviour

Variable	Standardized β	T value	P value	Standardized β	t value	P value
	Step 1			Step 2		
	Risky sexual			Disruptive		
	behaviour (RSB)			behaviour (DB)		
Peer pressure	.308	6.853	.000	.311	6.459	.000
Home environment						
(sub-scales)						
Responsivity	.308	6.853	.000	.311	6.459	.000
Encouragement of	.283	5.643	.000	.265	5.932	.000
maturity						
Emotional climate	.301	6.729	.000	.325	6.831	.000
Learning materials	.195	5.289	.000	.235	5.192	.000
Opportunities	−.202	−3.262	.001	−.233	3.501	.000
Enrichment	.294	4.689	.000	.278	4.701	.000
Family	.140	3.840	.001	.189	3.832	.000
companionship						
Physical	.289	5.871	.000	.275	6.091	.000
environment						

An examination of Table 1 shows a strikingly similar pattern in each model, whereby the peer pressure and the eight sub-scales of home environment were strong significant predictors of the disruptive and risky sexual behaviours. The results suggest that peer pressure and home environment exert a similar significant impact over disruptive and risky sexual behaviours. The results further suggest that stimulated home environment significantly predicts disruptive and risky sexual behaviours whilst unstimulated home environment has been found as a non-significant predictor of risky sexual behaviour and disruptive behaviour. Positive peer pressure on the other hand exerts a significant impact over the adolescents' risky sexual behaviour and disruptive behaviour whilst negative peer pressure has been found as a non-significant predictor of risky sexual behaviour and disruptive behaviour.

## Discussion

To the best of the researchers' knowledge, this is the first study in Enugu state, Nigeria. This study indicates that adolescents who reported their home environments to have high level of responsivity, encouragement of maturity, emotional climate, learning materials and opportunities, enrichment, family companionship and physical environment displayed compliant behaviour. Whilst those with chaotic and unstimulated home environment displayed disruptive behaviours such as interupting or intruding on others, arguing with others, lying to obtain favour or to avoid obligations, getting easily distracted by extraneous stimuli, engaging in physically dangerous activities without considering possible consequences, truant from school, violation of rules, initiating physical fights among others. The finding is in consonance with^13^ who explained that a home environment associated with parenting and family adversities is related to development and reinforcement of conduct problems. Parental psychopathology (especially a family history of conduct disorder/anti-social personality and substance use) are also related to an elevated risk for disruptive symptoms in offspring. Similarly, a study has shown that children raised in unstimulated or chaotic homes-characterized by noise, overcrowding, and lack of order, tend to score lower on tests of cognitive ability and self-regulatory capabilities, have poorer language abilities, and score higher on measures of problem behaviours such as disruptive disorder, conduct behaviour and learned helplessness than do children raised in stimulated or in a less chaotic environments^11^.

Furthermore, the present study found a strong and positive predictive power of home environment on adolescents' risky sexual behaviour. Also, stimulated home environment predicts adolescents with risky sexual behaviour, also adolescents' engagement in unprotected sex, multiple partners, being so drunk and being unable to resist sex, having sex without condom (unprotected sex), engaging in unexpected and unanticipated sexual experience among others, implicated risky sexual behaviour. The present finding is in line with Abert^22^ who noted that home environment has facilitated the development of risky sexual behaviours among young adults (and adolescents). In a similar vein,^7^ reported that a home environment with disruption of parents' marriage and a situation where the adolescent is living with a single parent associates with risky sexual behaviour, because there is seemingly less monitoring of the adolescents.

It was also found that peer pressure strongly predicts adolescents' DB. This finding demonstrates that adolescents who show a heightened sensitivity to negative peer pressure strongly exhibit disruptive behaviour. This finding agrees with Brendan^24^ that peer relationship difficulties have emerged as a salient and important predictor of mental health and behavioural adjustment. Adolescents with impaired peer relationships are at elevated risk of compounding problems in multiple domains of their life. Contrarily, adaptive peer relationships appear to buffer children, possibly through the development of positive social connections. The findings of this study reveal peer pressure as a strong predictor of adolescents' risky sexual behaviour. This finding indicates that adolescents with impaired or negative peer group have the tendency to engage in risky sexual behaviour while those that associate with positive or adaptive peer group are less vulnerable to risky sexual behaviour. This assertion was affirmed by Vaquera^18^ who asserted that a child influenced by a positive peer pressure can be inspired to be more focused and zealous, to share experiences and feelings and learn how to resolve conflicts, it provides adolescents with an appropriate environment for healthy development. Conversely, adolescents' relationship with negative peers, could lead to negative acts such as smoking, drinking, negative way of dressing and speaking, using illicit substances, engagement in sexual behaviours, adopting and accepting violence, adopting criminal and anti-social behaviours and in many other areas of the adolescent's life^19^.

Finally, these results support the evidence of the association between home/family, peer relationships and mental health, which may need to be considered when designing educational interventions, not only with the purpose of improving academic performance but also mental health among early adolescents.

## Conclusion

Adolescents' home environment has tremendous impact on their behaviour and life generally. In other words, if the home is not stimulated or is unable to provide the adolescent with basic necessities, the adolescent could be vulnerable to negative peer group pressure. However, unstimulated home environment and negative peer group could consequently interact to predispose these adolescents to disruptive behaviour and risky sexual behaviour. When these adolescents are subjected to such adverse behaviours by their unstimulated home environment and negative peers, it consequently becomes detrimental to their health, their parents/family and society at large.
